# Sleep disturbances in patients with multiple sclerosis

**DOI:** 10.1007/s10072-012-1229-0

**Published:** 2012-10-30

**Authors:** Anna Pokryszko-Dragan, Małgorzata Bilińska, Ewa Gruszka, Łukasz Biel, Katarzyna Kamińska, Katarzyna Konieczna

**Affiliations:** 1 Chair and Department of Neurology, Medical University of Wroclaw, Borowska 213, 50-556 Wroclaw, Poland; 2 Student Scientific Circle, Chair of Neurology, Medical University of Wroclaw, Wroclaw, Poland

**Keywords:** Multiple sclerosis, Sleep disturbances, Fatigue

## Abstract

Sleep disturbances constitute one of the important yet underestimated aspects of functioning of patients with multiple sclerosis (MS). The objective of this study was to evaluate sleep disturbances in patients with MS, with regard to demographic factors, disease-related variables, co-existing conditions and fatigue. In 100 MS patients, Epworth Sleepiness Scale (ESS) and a questionnaire about sleep disturbances (SlD) were implemented. ESS and SlD results were analyzed with regard to age, gender, duration of MS, type of its course, degree of disability in Expanded Disability Status Scale (EDSS), MS therapies, coexisting diseases, results of Fatigue Severity Scale (FSS) and Modified Fatigue Impact Scale (MFIS). ESS score indicated increased daytime sleepiness in 19 patients. In SlD, 49 subjects reported sleep disturbances and 35 more than one of their kind (most commonly terminal and middle insomnia). No relationships were found between ESS and SlD scores and age, gender, MS duration, type of its course, EDSS or coexisting diseases. In 36 patients, somatic complaints interfered with sleep. The patients with depression had significantly lower ESS result and those currently treated with immunomodulation had significantly lower SlD score. SlD score correlated positively with FSS and MFIS. Sleep disturbances in MS patients may occur independently from demographic and disease-related variables, but they are often influenced by the symptoms of MS and therapies used. Sleep disturbances may contribute to fatigue in the course of MS.

## Introduction

Multiple sclerosis (MS) is a chronic disease of the central nervous system, with disseminated inflammatory/demyelinative lesions and axonal loss leading to multifocal signs of neurological deficit. Recently, there has been growing interest in aspects of MS other than physical disability, which also seriously affect patients’ daily functioning and quality of life. Sleep disturbances, reported by 24–50 % of MS patients, constitute one such aspect [1–3]. Circadian rhythm disorders with compromised melatonin secretion [4], reduced input to the suprachiasmatic nucleus due to impaired visual pathways [5] as well as increased levels of proinflammatory cytokines [6] have been suggested as possible pathomechanisms shared by MS and sleep disturbances. However, problems with sleep may be also associated with symptoms and signs of the disease, side effects of MS therapies, psychological problems secondary to the disease (including depression and anxiety) or other diseases coexisting with MS. Thus, the background of sleep disturbances in MS patients seems complex and multifactorial.

In MS literature, there are relatively few original studies focusing on sleep problems in this group of patients [7–10]. Sleep disturbances have mainly been discussed with regard to fatigue: a common clinical manifestation of MS, with a serious impact upon patients’ quality of life [4, 5, 11–14]. Studies on this subject are based on a variety of methods and mostly include small groups of patients.

The main purpose of our study was to evaluate the prevalence and type of sleep disturbances in MS patients, with regard to demographic factors, disease-related variables and co-existing conditions. We also aimed to investigate relationships between sleep disturbances and fatigue.

## Methods

The study comprised 100 patients (31 men and 69 women, aged 20–67 years, mean 42 years) with clinically definite MS, according to McDonald’s criteria [15]. The patients were under the care of the MS outpatient clinic at the Department of Neurology, Medical University of Wroclaw. All the patients gave their informed consent to participate in the study and the project was approved by the local Bioethics Committee at Medical University of Wroclaw.

On the basis of interview and neurological examination, the current degree of the patients’ disability was assessed in the Expanded Disability Status Scale (EDSS) [16]. On the basis of medical records, duration of MS and course type were established, as well as disease-modifying and symptomatic treatment due to MS, coexisting conditions and medications used in their regard. The Polish validated version of Epworth Sleepiness Scale (ESS) [17, 18] was used to assess daytime sleepiness. For more detailed evaluation of sleep problems, a questionnaire of our authorship was implemented. Its first part consisted of 14 questions concerning the following problems: difficulties with falling asleep (initial insomnia), awakenings during the night (middle insomnia), snoring, dyspnea, nightmares and behavioral disturbances during sleep, waking up early in the morning without falling asleep again (terminal insomnia), difficulties with waking up in the morning, the feeling of inefficient night sleep, the need for naps during the day, irregularities of evening and morning sleep schedules, somatic complaints and anxiety interfering with sleep. Depending on the frequency of particular problems, the subjects could score 0–4 for each of the statements. The sum of these scores was calculated as a total result (named SlD—sleep disturbances), used in the further analysis. Validation of this part of questionnaire has been conducted in our Department. In the second part of the questionnaire, the patients were asked to describe symptoms of MS which affect their sleep and to name methods they use to relieve their problems with sleep. Level of fatigue was assessed using the Polish validated versions of Fatigue Severity Scale (FSS) [19] and Modified Fatigue Impact Scale (MFIS) [20, 21].

We investigated correlations between the ESS and SlD results and age, duration of MS and EDSS. ESS and SlD results were compared between genders and between subgroups of patients with different MS course types (relapsing–remitting vs. secondary/primary progressive), with coexisting diseases or without them and treated with immunomodulation or not.

To evaluate relationships between sleep disturbances and fatigue, ESS and SlD were compared between non-fatigued and fatigued MS patients (FSS ≤ 3.5 or >3.5, respectively). FSS and MFIS results were compared between the patients who scored 0–2 versus 3–4 in the statements of SlD concerning initial, middle and terminal insomnia, as well as problems with waking up in the morning and the feeling of inefficient sleep. Correlations were also investigated between ESS and SlD and the fatigue measures (FSS, MFIS, subscales of MFIS).

Statistical analysis was carried out with the use of STATISTICA (StatSoft). *p* = 0.05 was considered as a basic significance level. To examine the differences between two independent continuous variables, Mann–Whitney *U* test was used. Pearson’s correlation coefficient was computed to detect the relationship between the continuously normally distributed variables. If the normality assumption was not met, the Spearman rank correlation coefficient was used.

## Results

The duration of MS in study group ranged from 1 to 36 years (mean 8.6) and EDSS was 1.0–7.5 (mean 3.2). 79 patients presented with a relapsing–remitting course of MS (RR-MS), 16 with a secondary progressive (SP-MS) and 5 with a primary progressive (PP-MS) course. Thirty-two patients were currently being treated with interferon beta (IFNβ) and one with glatiramer acetate (GA). None of the patients were interviewed during either a relapse or treatment with corticosteroids.

In 13 patients, coexisting diseases were recognized: heart diseases in 7, hypothyroidism in 4, and chronic nephritis in 2. None of the patients were diagnosed with obstructive sleep apnea syndrome or other sleep-related disorders. Thirteen subjects had been diagnosed with depression. Forty-six subjects were using medications which can cause sleepiness as a side effect (miorelaxants, cholinolytics, carbamazepine or gabapentine for the treatment of neuropathic pain, antidepressants).

ESS results ranged from 0 to 19 (mean 6.27). 81 patients scored <10 on the ESS, 15 patients obtained 10–16 points, which correspond to excessive sleepiness, and 4 subjects scored more than 16 points (pathological sleepiness). Total SlD scores were 1–23 (mean 7.99). Answering SlD questionnaire, 49 patients reported sleep disturbances; 35 subjects reported more than one of their kind; 48 patients reported terminal insomnia (waking up too early); 33, middle insomnia (awakenings during the night); 28, initial insomnia (difficulties with falling asleep); 18, difficulties with getting up in the morning; 17, subjective feeling of inefficient sleep; 13, snoring during sleep (2 of them experienced dyspnea at night); and 9 complained of nightmares. In 36 cases, somatic complaints due to MS interfered with sleep; in 4 subjects, problems with sleep were associated with anxiety.

The ESS results showed significant positive correlations with the SlD score (*r* = 0.31, *p* = 0.01). There were no correlations between ESS and SlD results and the age of the subjects. The mean ESS and SlD results did not differ significantly between male and female patients (Table 1). No significant correlations were found between ESS or SlD results and duration of MS or EDSS. No differences in mean ESS and SlD results were found between the patients with RR-MS and those with a progressive course of the disease (SP-MS and PP-MS) (Table 1).

**Table 1:** Relationships between ESS and SlD results in patients with MS and gender, disease-related variables and coexisting conditions

	ESS			SlD		
	Mean	SD	*p*	Mean	SD	*p*
Gender						
Men (*n* = 31)	5.58	3.74	0.23	6.62	4.4	0.15
Women (*n* = 69)	7.74	5.04		8.13	5.26	
MS course						
RR-MS (*n* = 79)	5.99	3.93	0.42	7.81	5.17	0.52
SP/PP-MS (*n* = 21)	7.33	5.31		8.67	5.26	
Immunomodulating treatment						
Yes (*n* = 34)	6.09	4.08		6.24	4.29	**0.02**
No (*n* = 66)	6.62	4.37		8.89	5.38	
Coexisting diseases						
Yes (*n* = 23)	6.13	4.96	0.58	8.74	6.06	0.67
No (*n* = 77)	6.31	4.07		7.77	4.09	
Depression						
Yes (*n* = 13)	3.92	3.62	**0.01**	10.69	7.12	0.11
No (*n* = 87)	6.62	4.26		7.59	4.74	
Fatigue						
Yes (FSS > 3.5) (*n* = 49)	6.35	3.93	0.56	6.06	3.92	**0.0001**
No (FSS ≤ 3.5) (*n* = 51)	6.18	4.67		10.0	5.58	

*ESS* Epworth Sleepiness Scale, *FSS* Fatigue Severity Scale, *MS* multiple sclerosis, *PP-MS* primary progressive multiple sclerosis, *RR-MS* relapsing–remitting multiple sclerosis, *SD* standard deviation, *SlD* sleep disturbances (the questionnaire score), *SP-MS* secondary progressive multiple sclerosis

Statistically significant level of *p* < 0.05 was highlighted with bold

Among MS symptoms which influenced sleep quality, 24 patients reported spasticity with muscle cramps or uncontrolled limbs movement (clonus), 23 pain or paresthesiae and 17 bladder dysfunction (urinary urgency or incontinence, nocturia). Answering particular parts of the SlD questionnaire, 46 % of the patients with spasticity-related symptoms reported frequent (score 3–4) initial insomnia, 33 % with middle insomnia and 20 % with terminal insomnia (those with pain or paresthesiae—48, 36 and 32 %, respectively, and those with bladder dysfunction—36, 53 and 47 %, respectively).

Patients diagnosed with depression had significantly lower ESS scores than the remaining ones, but they did not differ in their SlD scores. No significant differences in ESS and SlD results were found between subgroups of patients with or without other diseases coexisting with MS (Table 1).

Patients currently being treated with immunomodulating agents had significantly lower SlD scores than the remaining ones but no such differences were found for ESS (Table 1). Among the patients undergoing such treatment, 14 (41 %) reported terminal insomnia; 10 (29 %), middle insomnia; 8 (24 %), initial insomnia; and 2 (6 %), difficulties with waking up in the morning.

To relieve their sleep disturbances, 23 patients used hypnotic agents: benzodiazepines, zolpidem or zopiclone (10 subjects, regularly; 13, sporadically), 10 patients used alternative methods (herbal mixtures, yoga, relaxation techniques), 8 undertook relaxing activities: reading, watching TV, listening to music and 6 drank warm beverages (tea, milk).

FSS results ranged from 1.1 to 7.0 (mean 3.8); MFIS from 1 to 75 (mean 33.8). Non-fatigued MS patients (FSS ≤ 3.5) had significantly lower SlD scores than fatigued ones, while no such differences were found for ESS (Table 1). No correlation was found between ESS and fatigue measures (FSS or MFIS), while SlD scores correlated significantly with both FSS and MFIS results (*r* = 0.43, *p* = 0.001 and *r* = 0.62, *p* = 0.001, respectively). There were also significant correlations between SlD and all the subscales of MFIS: physical, cognitive and social (*r* = 0.58, *p* = 0.001; *r* = 0.5, *p* = 0.001; *r* = 0.53, *p* = 0.001, respectively). Among particular sleep disturbances assessed in the SlD, patients with initial insomnia (score 3–4) had significantly higher fatigue measures than the remaining ones (FSS: 4.43 ± 1.67 vs. 3.58 ± 1.62, *p* = 0.02; MFIS: 41.5 ± 19.76 vs. 30.81 ± 17.84, *p* = 0.02). The same was the case with patients with middle insomnia (FSS: 4.51 ± 1.61 vs. 3.47 ± 1.6, *p* = 0.02; MFIS: 41.73 ± 18.25 vs. 29.9 ± 18.13, *p* = 0.001) and a feeling of insufficient sleep in the morning (FSS: 5.07 ± 1.4 vs. 3.52 ± 1.6, *p* = 0.001; MFIS: 51.95 ± 13.8 vs. 29.54 ± 17.4, *p* = 0.001). Patients reporting difficulties with waking up in the morning have significantly higher MFIS scores (44.39 ± 21.1 vs. 31.48 ± 17.7, *p* = 0.01) without differences in FSS. The results of FSS and MFIS did not differ significantly between subgroups of patients with or without other sleep disturbances evaluated in the SlD.

## Discussion

Almost 50 % of the MS patients in the studied group complained of sleep disturbances, with 35 % reporting more than one kind.

The results of ESS in 15 % of our patients corresponded to excessive sleepiness and only in 4 % with pathological sleepiness. Merkelbach et al. [22] obtained similar data, while both lower and higher values were noted by other authors [12, 13]. A significant correlation between ESS and SlD indicates the consistency of these measures in the evaluation of sleep problems. The most frequent disturbances reported by the patients included terminal insomnia, followed by middle and initial. Waking up too early has been described as a common problem of the MS patient [2, 13]. Although Attarian et al. [23] observed misperception of sleep in MS patients, their subjective complaints of problems with falling asleep and awakenings during the night were confirmed by the results of the Multiple Sleep Latency Test [5], polysomnography [24] and actigraphy [11].

We did not find relationships between ESS or SlD and demographic factors. The data on that subject are scarce. Merkelbach et al. [14] also found a lack of correlation between ESS and age. Bamer et al. [1, 10] suggested a higher prevalence of sleep disturbances in female patients, while Hossein et al. [25] found only lower alertness score in women, with less frequent sleep pathology.

In our study, sleep disturbances did not depend on disability, duration of disease or its course. Some studies suggested that higher EDSS and shorter MS duration are associated with a greater prevalence of sleep disorders [1, 26], but the majority of authors [7, 8, 13, 14] did not find such relationships. However, 40 % of our subjects reported symptoms of MS affecting their sleep. Spasticity and pain or paresthesiae interfered mainly with falling asleep and continuous night sleep. Bladder dysfunction contributed equally to all types of insomnia. Observations concerning pain and nocturia are consistent with the data from other studies [7, 13, 24]. Spasticity-related symptoms and paresthesiae need to be differentiated from restless legs syndrome (RLS), which is estimated to occur in up to 37 % of MS patients [9, 12, 27]. Symptoms of RLS were not specifically addressed in our questionnaire, but the descriptions of complaints seem unspecific for this syndrome (muscle cramps, increased muscle tone, clonus, tingling or burning of various localisations: not limited to the legs, without mentioning the urge to move the limbs). It is worth considering that medications used to relieve the above MS symptoms (miorelaxants, analgesics, cholinolytics) could diminish their impact upon sleep, but also cause sleepiness as a side effect.

Apart from heart diseases, which in two cases were associated with dyspnea at night, coexisting diseases (recognized only in 13 % of our patients) did not significantly influence sleep disturbances. None of the patients was diagnosed with sleep-related breathing disorders. Although their risk was suggested to increase with the presence of demyelinative lesions in the brainstem, polysomnographic studies did not confirm their greater frequency in MS patients or the relationship between daytime sleepiness and episodes of nocturnal apnea or oxygen desaturation [12, 28, 29].

Depression and anxiety are usually regarded as important confounding factors in sleep disturbances in MS patients [2, 7, 9, 13, 30]. Depression (recognized in 13 % of our patients) was rather unexpectedly associated with lower daytime sleepiness, without influence upon sleep characteristics evaluated by SlD. All these patients were being treated with antidepressants, which might also improve their sleep quality. Only four subjects reported anxiety contributing to initial insomnia, so it was not possible to analyze this relationship statistically. Assessment of depression and anxiety, using appropriate neuropsychological tools, may be useful in the complex evaluation of sleep disturbances in MS patients.

We found a lower prevalence of sleep disturbances in MS patients undergoing immunomodulating treatment, without differences in daytime sleepiness. Problems with sleep (insomnia or hypersomnia) are described among the side effects of treatment with IFN-β and GA [2, 31]. However, our findings seem to support the evidence that IFN-β might ameliorate sleep disturbances in MS, upregulating their possible background: an increase in the level of cytokines or disrupted circadian secretion of melatonin with its suppressed metabolism [3, 6].

The relationships between sleep disturbances and fatigue remain a matter of debate. Sleep problems are regarded as a predictive factor and contributor to fatigue. Despite a partial overlap between these entities, they should not be coalesced [3, 11–14, 21, 25, 32]. We found no differences in the level of sleepiness between fatigued and non-fatigued MS patients and no correlations between ESS and both fatigue measures. These findings, consistent with the results of Kaynak et al. [12], support the concept that excessive sleepiness and fatigue are distinct conditions. Other authors [11, 14, 22], who found more significant relationships between ESS and FSS, suggested that sleepiness and fatigue may converge for some situations (especially involving the subject’s active approach) but differ in other circumstances. Unlike ESS, the SlD score was significantly higher in our fatigued MS patients and correlated with both fatigue measures and all the subscales of MFIS. Thus, sleep disturbances affected physical, cognitive and social aspects of fatigue. Initial and middle insomnia showed the most significant relationships to fatigue. In studies on that subject, the link between fatigue and middle insomnia is stressed. Recurrent arousals from sleep, with disruption of sleep microstructure shown in polysomnography [12, 13], are suggested to cause excessive CNS activation—one of the hypothesized pathomechanisms of fatigue [3, 32].

In our study, <25 % of subjects used hypnotic agents (and only 10 % took them regularly). Bamer et al. [10] found that up to 30 % of a MS cohort used sleep medications (including 19 % regular users). The lower prevalence of pharmacological agents use in our MS patients may suggest an underestimation of the sleep problems by their physicians or perhaps a trend to avoid sedative medications. Only 10 % of the studied group used alternative therapies which are, otherwise, popular among MS patients. Altogether, problems with sleep seemed not appropriately handled in this group of patients.

In conclusion, sleep disturbances constitute a serious problem and deserve more attention in MS patients. They may occur independently from demographic factors and disease-related variables. However, the quality of sleep is influenced by the symptoms of neurological deficit as well as medications used in the treatment of MS. Sleep disturbances may contribute to fatigue in the course of MS.
